# Synthesis and Application of a Glucoconjugated Organometallic Rhenium Complex as an IR Imaging Probe for Glycolytic Cancer Cells

**DOI:** 10.3390/molecules31010028

**Published:** 2025-12-22

**Authors:** Giulia Bononi, Erica Paltrinieri, Serena Fortunato, Gaspare Cicio, Nicola Di Giovanni, Giulia Lencioni, Niccola Funel, Elisa Giovannetti, Carlotta Granchi, Valeria Di Bussolo, Filippo Minutolo

**Affiliations:** 1 Department of Pharmacy, University of Pisa, Via Bonanno 6, 56126 Pisa, Italy; giulia.bononi@unipi.it (G.B.); serena.fortunato@unipi.it (S.F.); carlotta.granchi@unipi.it (C.G.); valeria.dibussolo@unipi.it (V.D.B.); 2 Center for Instrument Sharing of the University of Pisa (CISUP), Lungarno Pacinotti 43, 56126 Pisa, Italy; 3 Cancer Pharmacology Laboratory, AIRC Start-Up Unit, Fondazione Pisana per la Scienza, Via Ferruccio Giovannini, 13, San Giuliano Terme, 56017 Pisa, Italy; g.lencioni@fpscience.it (G.L.);; 4 USL Tuscany Northwest Location Lucca, Department of Laboratory Diagnostics, Via Lippi Francesconi, 55100 Lucca, Italy; niccola.funel@uslnordovest.toscana.it; 5 Department of Medical Oncology, Cancer Center Amsterdam, Amsterdam University Medical Center, Vrije Universiteit Amsterdam, 1081 HV Amsterdam, The Netherlands

**Keywords:** metal-carbonyl complexes, glucoconjugates, glucoconjugated rhenium complexes, rhenium tricarbonyl complexes, cancer imaging, infrared (IR) probes

## Abstract

Current tumor diagnostics rely on fluorodeoxyglucose (FDG)-PET imaging, but FDG’s short half-life and high cost limit its widespread use. Infrared (IR) probes are emerging as non-radioactive alternatives to conventional tracers for tissue section and other in vitro imaging applications. Because cells and tissues are relatively free of absorption peaks between 1800 and 2200 cm^−1^, metal-carbonyl complexes, especially cyclopentadienylrhenium(I) tricarbonyl (Cp[Re(CO)_3_]) derivatives, absorb strongly in this window and provide robust platforms for bioconjugation. Furthermore, Cp[Re(CO)_3_] fragments can be introduced into organic substrates via an elegant three-component reaction that simultaneously forges the cyclopentadienyl-metal and cyclopentadienyl-substituent bonds. As a result, the functionalized half-sandwich complex is obtained in a single step without any special handling issues. We have therefore properly modified a glucose molecule with that complex and developed a novel glucoconjugated Cp[Re(CO)_3_] probe that enables IR-based visualization of diseased cells at 2100 cm^−1^, offering a non-invasive, non-radioactive histological tool and a promising basis for future medical imaging devices.

## 1. Introduction

**Figure 1:**
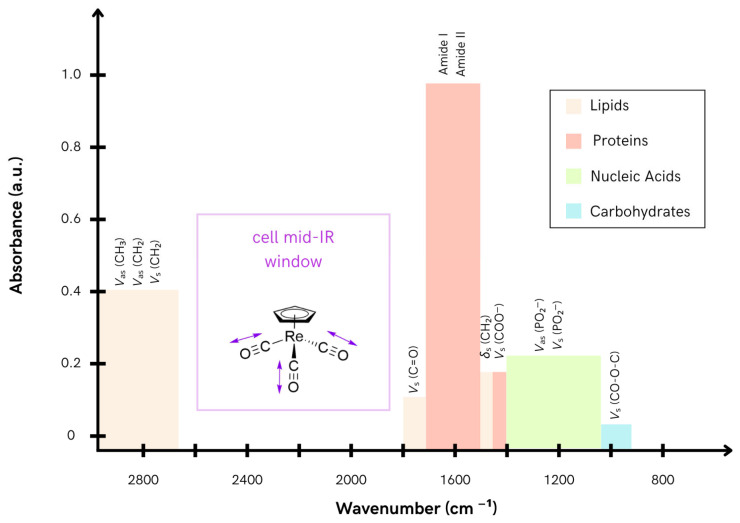
Infrared absorption peaks of the most relevant biomolecules present in cells, namely, lipids, proteins, nucleic acids and carbohydrates, and the mid-IR window that can be exploited for IR imaging by Cp[Re(CO)_3_] complexes, which display very strong symmetrical and asymmetrical stretching absorptions in that wavenumber range.

The development of infrared (IR) probes is an emerging field, particularly in medical diagnostics, which currently relies primarily on radioactive or radiopaque probes, luminescent probes, and other similar technologies. In contrast, diagnostic methods based on IR spectroscopy are not yet widely adopted. It is well known that cells and tissues exhibit minimal absorption in the IR window between 1800 and 2200 cm^−1^, making this frequency range suitable for diagnostic and physicochemical characterization (Figure 1) [1]. This can be achieved using probes with strong absorption properties within this range, conjugated with biomolecules to facilitate interactions with specific biological targets, although it should be acknowledged that IR imaging necessarily has some drawbacks due to the limited penetration depth (≤100 µm) of mid-IR radiation.

Chemical compounds with strong absorption peaks in this range are predominantly found among metal-carbonyl complexes [2]. These complexes offer higher sensitivity than other functional groups commonly used in this field (such as nitrile groups) due to their high extinction coefficient, often exceeding 4000 M^−1^cm^−1^. Consequently, they have garnered increasing interest as potential IR probes. Among them, cyclopentadienylrhenium(I) tricarbonyl (Cp[Re(CO)_3_]) complexes stand out as particularly promising candidates, given their strong IR absorption in the 1900–2100 cm^−1^ range and their favorable chemical and structural properties for diagnostic applications. We selected rhenium tricarbonyl complexes over other carbonyl-coordinated transition-metal options because of their reportedly superior stability, even under harsh conditions, particularly when they are part of organometallic complexes containing a coordinating cyclopentadienyl unit. In fact, these complexes are chemically stable, resistant to hydrolysis in aqueous environments, and not subject to rapid enzymatic degradation in biological systems [3]. Additionally, their small size and predominantly hydrophobic nature help minimize interference with the interactions necessary for conjugation to biomolecules and subsequent binding to biological targets. For example, Cp[Re(CO)_3_] moieties have already been conjugated to large proteins to label them and make them IR-visible in aqueous solutions [4]. Glucose-conjugated inorganic rhenium tricarbonyl complexes containing bidentate amine ligands have previously been reported [5], although the resulting molecules were found to exhibit some instability due to displacement of the heteroatom ligand from the metal center. In contrast, highly stable glycoconjugated organometallic cyclopentadienyl-based *fac*-[Re(CO)_3_] complexes have not yet been reported in the literature, to the best of our knowledge.

An augmented uptake of glucose is a characteristic feature of various cancers, including pancreatic cancer [6]. As tumors grow rapidly, cancer cells often experience hypoxia, leading them to rely primarily on glycolysis for energy production [7]. However, glycolysis yields only two molecules of adenosine triphosphate (ATP) per glucose molecule, whereas complete oxidative phosphorylation produces 36 ATP molecules. To compensate for the low energy yield under hypoxic conditions, cancer cells upregulate glucose transporters (GLUTs), to ensure sufficient energy production and biomass synthesis. Due to its pivotal role in tumor metabolism, enhanced glucose uptake has become a key therapeutic and diagnostic factor [8]. Furthermore, glucoconjugated metal complexes have already been reported as perspective anticancer agents [9] including some novel ruthenium(II) arene complexes bound to a single glucose unit [10].

Radiolabeled glucose analogs are being clinically utilized as diagnostic probes. In fact, tumor detection and characterization currently rely predominantly on radioactive markers such as fluorodeoxyglucose (FDG), commonly used in positron emission tomography (PET) [11]. FDG, a radioactive glucose analog, enables visualization of cancerous tissues by exploiting the distinct metabolic activity of tumors, which rapidly absorb large amounts of glucose. While this imaging technique is widely utilized in vivo, it is less suited for routine applications requiring tumor cell identification in ex vivo samples obtained from solid or liquid biopsies. A primary limitation of FDG-based diagnostics is the reliance on fluorine radionuclide (^18^F), which has a relatively short half-life of approximately two hours. As a result, FDG must be synthesized using specialized generators, which are not always available near diagnostic facilities. Additionally, radiopharmaceuticals like FDG require stringent safety precautions during production and handling, leading to significantly increased costs for laboratory and healthcare personnel.

We herein report a specific modification of a β-glucopyranosyl unit leading to the synthesis of a new Cp[Re(CO)_3_] complex conjugated to the sugar moiety to promote its uptake in cancer cells. Although this approach should currently be limited only to isolated cell cultures or thin tissues, due to the lower penetration depth of infrared signals compared to those emitted by radiopharmaceuticals such as FDG, this complex exhibits strong IR absorption within the minimal IR absorption window of the targeted cells, enabling their visualization through an infrared microscope at a peculiar IR spectroscopy frequency.

## 2. Results and Discussion

### 2.1. Design, Synthesis and Characterization of Rhenium Cyclopentadienyl Glucoconjugate 11

**Figure 2:**
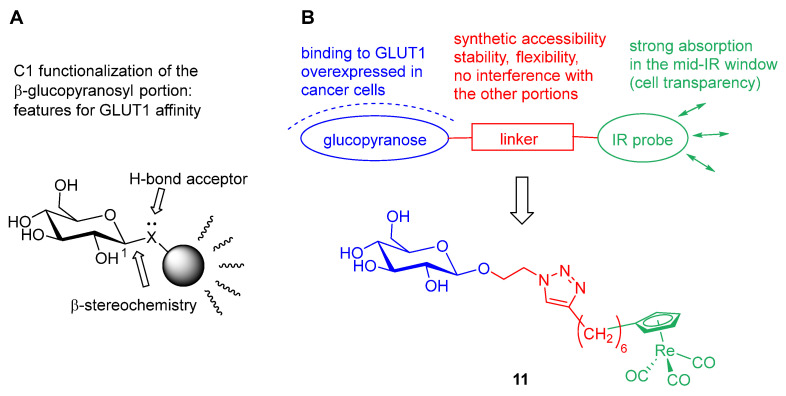
Panel (**A**): Modifications of the β-glucopyranosyl moiety at the C1 position that allow the resulting conjugate to retain its ability to be taken up by cells [12]. Panel (**B**): Structure of the designed infrared probe **11**; the biorelevant portion (i.e., β-glucopyranose) is highlighted in blue, the linker in red, and the Cp[Re(CO)_3_] moiety in green.

The IR-probe design included several components: (a) a glucose unit to facilitate accumulation in cancer cells [12]; (b) a molecular moiety displaying a strong IR absorption in the cellular minimal IR-absorption window (1800 and 2200 cm^−1^) to avoid interference by cellular components; (c) a suitable linker connecting the two aforementioned parts (Figure 2).

The β-glucopyranose unit was chosen as a glucose mimic, as it has been widely shown to be potentially recognized by the glucose transporters (Figure 2A) [12]. For example, researchers have applied this approach in discovering newly approved gliflozins, where a β-glycosidic linkage at C1 of the glucose moiety ensures efficient engagement with sodium-glucose cotransporters [13]. As far as the linker is concerned, a variety of possible linkers have already been explored in the design of β-glucopyranose conjugates [14]. We employed a linker chosen for synthetic accessibility, reagents availability (1,7-octadiyne) and minimal disruption of sugar-protein binding, incorporating a triazole ring introduced by click chemistry [15], attached to a saturated flexible alkyl chain that should prevent interference of the aglycone portion with the binding process of the glucose unit. Finally, the IR-active portion was identified in a Cp[Re(CO)_3_] core. In fact, this organometallic complex is chemically and metabolically stable [3], enabling easy handling and efficient cellular distribution. Additionally, its lipophilic nature and compact size minimize interference with the probe’s binding process, whether due to polarity or steric hindrance. Most importantly, the Cp[Re(CO)_3_] complex exhibits very favorable vibrational properties, with two distinct and intense absorption bands within the cellular minimal-IR absorption window (approximately 2030 and 1900 cm^−1^, respectively, corresponding to the asymmetric and symmetric stretching vibrations of the three CO bonds). This property facilitates the detection of the designed complexes within cellular environments. We therefore planned the site-specific stereoselective functionalization of the glucose unit, leading to the synthesis of a new glucoconjugated Cp[Re(CO)_3_] complex (compound **11**, Figure 2).

**Scheme 1:**
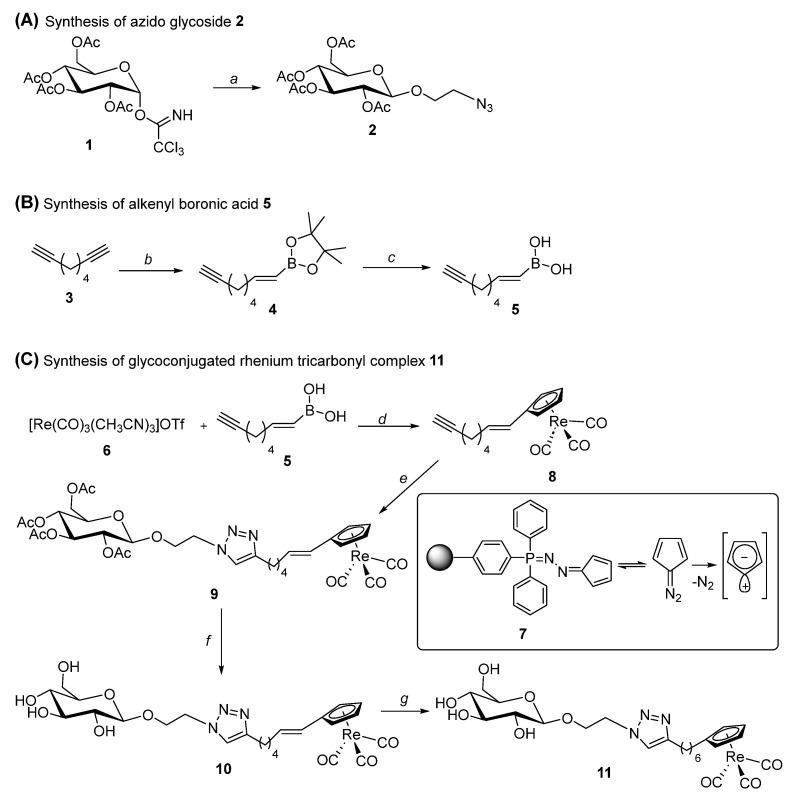
Synthesis of Cp[Re(CO)_3_] glycoconjugate **11**. *Reagents and conditions*: (*a*) 2-azidoethanol, TMSOTf, AW-300, dry CH_2_Cl_2_, from −20 °C to RT, 24 h [45%]; (*b*) pinacolborane, [Fe_2_(CO)_9_], dry toluene, 100 °C, 16 h [32%]; (*c*) NaIO_4_, NH_4_OAc, acetone/H_2_O 1:1 *v*/*v*, RT, 16 h [92%]; (*d*) polymer supported-PPh_2_N_2_Cp **7** (3.0 mmolP/g, 80% functionalized), Et_3_N, dry CH_3_CN, 80 °C, 16 h [14%]; (*e*) **2**, *t*-BuOH/1,4-dioxane 1:1 *v*/*v*, sodium ascorbate in H_2_O, CuSO_4_·5H_2_O in H_2_O, 100 °C, 20 h [80%]; (*f*) MeONa (s), dry MeOH, RT, 51 h [83%]; (*g*) H_2_ (1 atm), 10% Pd/C, MeOH, RT, 16 h [79%].

The general synthetic pathway to obtain the desired glucoconjugated Cp[Re(CO)_3_]probe **11** is reported below in Scheme 1.

The glycosylation step between compound **1** [16] and 2-azidoethanol [17] was carried out in the presence of molecular sieves AW-300 as the drying agent and anhydrous dichloromethane as the solvent (A, Scheme 1). The subsequent activation of glycosyl donor **1** using a solution of trimethylsilyl trifluoromethanesulfonate (TMSOTf) as the catalyst led to the formation of *tetra*-acetylated derivative **2** in a completely β-stereoselective manner and in good yields (step *a*, Scheme 1).

The second part of the synthesis involves the formation of alkenyl boronic acid **5** starting from its pinacolborane ester precursor **4** (B, Scheme 1). (*E*)-4,4,5,5-tetramethyl-2-(pent-1-en-4-yn-1-yl)-1,3,2-dioxaborolane **4** was synthesized by an iron-catalyzed hydroboration reaction between commercially available 1,7-octadyine (**3**) and pinacolborane in anhydrous toluene at 100 °C for 16 h, yielding the desired intermediate **4** (step *b*, Scheme 1).

Next, the oxidative cleavage of pinacolborane ester **4** to the corresponding boronic acid **5** was performed using sodium metaperiodate (NaIO_4_) and ammonium acetate (NH_4_OAc) in a 1:1 (*v*/*v*) acetone/water mixture (step *c*, Scheme 1), yielding **5** in very high yields.

Boronic acid **5** was chosen as the key intermediate due to the proven good reactivity of this type of carbon nucleophile in forming substituted Cp[Re(CO)_3_] complexes through a three-component reaction with diazocyclopentadiene and the proper *fac*-[Re(CO)_3_] (**6**, Scheme 1) precursor in acetonitrile at 80 °C. Indeed, previous studies from our research group demonstrated that boronic acids act as efficient “masked-carbanions” under these conditions, thus efficiently promoting the C-C bond formation with the Cp ring [18]. Polymer-bound diazocyclopentadiene **7** (Scheme 1) was later preferred to its soluble counterpart since it is stable under ambient conditions, including exposure to humidity and light [19]. In acetonitrile it dissociates rapidly into polymer-bound triphenylphosphine (PPh_3_) and free diazocyclopentadiene, which then participates in the three-component reaction (step *d*, Scheme 1). Finally, the resulting polymer-bound PPh_3_ can be easily removed by simple filtration from the reaction mixture. Therefore, boronic acid **5**, diazocyclopentadienyl resin **7** and [Re(CO)_3_(CH_3_CN)_3_]OTf **6** were subjected to a one pot three-component reaction to yield Cp[Re(CO)_3_] complex **8** (step *d*, Scheme 1).

The diazocyclopentadienyl nucleus was generated in situ by heating a solution of phosphazine **7** in dry acetonitrile at 80 °C. Upon formation, diazocyclopentadiene quickly releases molecular nitrogen, acting as a bifunctional synthon facilitating the nucleophilic substitution at its vacant σ-orbital while simultaneously donating six π-electrons of the Cp ring to the [Re(CO)_3_]^+^ core (inbox, Scheme 1). Consequently, diazocyclopentadiene underwent concurrent attack by [Re(CO)_3_]^+^ and vinyl-boronic acid **5**, leading to the formation of the desired complex **8**.

At this stage, Cu(I)-catalyzed Huisgen 1,3-dipolar cycloaddition between azides **2** and terminal alkyne-functionalized Cp[Re(CO)_3_] complex **8** yielded the final glucoconjugated Cp[Re(CO)_3_] complex **9** in high yields (step *e*, Scheme 1). Deacetylation of the glucose portion of **9** using solid MeONa in MeOH efficiently produced intermediate complex **10** (step *f*, Scheme 1). Finally, the desired complex **11**, containing a fully saturated alkyl chain between the triazole and the cyclopentadienyl ring, was obtained in high yields by hydrogenation of the C5–C6 double bond of compound **10** in the presence of Pd/C (10%) as the catalyst (step *g*, Scheme 1).

**Figure 3:**
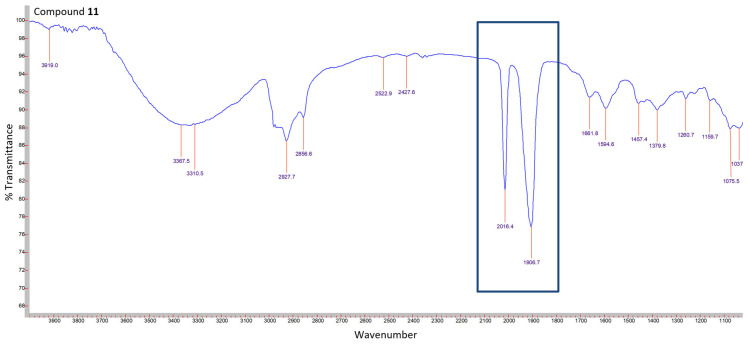
IR spectrum of final glucoconjugated complex **11** (solid) displaying the two typical peaks at about 2020 and 1910 cm^−1^ of Cp[Re(CO)_3_] complexes, which are properly placed within the cell mid-IR window.

Before proceeding with the imaging assays, newly synthesized Cp[Re(CO)_3_] glucoconjugate **11** was fully characterized by nuclear magnetic resonance (^1^H-and ^13^C-NMR), high-resolution mass spectrometry (HRMS) analysis, and its purity was found to be 95% by high performance liquid chromatography (HPLC) analysis. Additionally, IR spectrum of **11** (Figure 3) confirmed that glucoconjugation of the Cp[Re(CO)_3_] unit did not affect its peculiar and very strong absorption bands at 2020 and 1910 cm^−1^ respectively due to symmetrical and asymmetrical stretching of the three CO metal ligands.

### 2.2. In Vitro FTIR Microscope Imaging and Detection of Pancreatic Ductal Adenocarcinoma (PDAC) Cells

**Figure 4:**
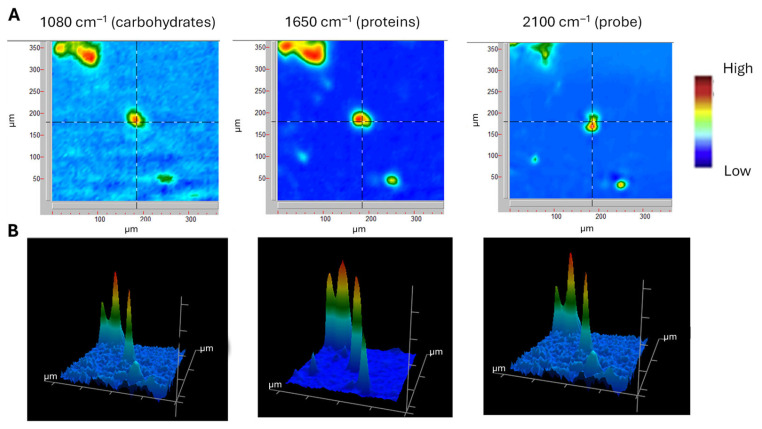
PDAC cells treated with compound **11**: 2D IR absorption intensities at the indicated wavelength (**A**); 3D visualization of IR absorption peaks at the indicated wavelength (**B**).

Primary cultures of pancreatic tumor cells (PCC-PDAC) were selected since they are notoriously highly glycolytic. Notably, quantitative PCR analysis revealed that GLUT1 expression levels in these cells were over five times higher than those observed in the normal immortalized pancreatic ductal cells HPDE kindly supplied by Dr. Tsao [20]. GLUT1 may not be the only transporter involved in the uptake of our complex; nevertheless, its overexpression is generally considered to be characteristic of glycolytic cancer cells. These cells were immobilized on trans-reflectance MirrIR slides and exposed to 100 μM of glucoconjugated complex **11** for 1 h. Complex **11** remained stable under these experimental conditions throughout the incubation (Figures S6–S8). Furthermore, no significant cytotoxicity was observed under these incubation conditions (Figure S10). Afterwards, the slides were examined with the FTIR Microscope fitted with a liquid nitrogen-cooled focal plane array (FPA) detector. Each analysis was performed in reflectance mode with 64 scans for both the samples and the background, at a resolution of 4 cm^−1^. Images were acquired at the two characteristic IR absorption frequencies of carbohydrates (1080 cm^−1^) and proteins (1650 cm^−1^), and at 2100 cm^−1^, the distinct wavenumber where the Cp[Re(CO)_3_] complex exhibits its maximal absorption (Figure 4). In these maps, the x and y axes represent the spatial coordinates of the scanned slide area. Panel A (Figure 4) displays the 2D spatial distribution of IR absorption intensities, whereas Panel B (Figure 4) shows the corresponding 3D visualization of these data. The regions of the slide identified by carbohydrate and protein signals, corresponding to the locations of cancer cells, closely coincide with the areas of strongest rhenium-complex absorption. In more detail, cell-containing regions showed an average reduction in transmittance at 2100 cm^−1^ of about 40% relative to the background.

It should be noted that unsubstituted Cp[Re(CO)_3_] did not exhibit the same specific accumulation in the region of the microscope slide occupied by PDAC cells. Instead, it remained broadly distributed across the slide, likely due to its relatively high lipophilicity and consequent poor aqueous solubility, which hindered efficient rinsing prior to measurement (Figure S9). The comparison with unsubstituted Cp[Re(CO)_3_] is indeed valuable, as it better highlights how this control supports the role of the glucose unit in improving association with the cells and the subsequent washability of the unbound compound. These observations support the hypothesis that glucoconjugated complex **11** accumulates in regions containing pancreatic cancer cells, thereby confirming that this probe enables their visualization by FTIR microscopy. However, due to the diffraction-limited spatial resolution of this technique (as defined by the Abbe criterion), it is not currently possible to determine whether the complex is internalized within the cancer cells or remains associated with the cell membrane where glucose transporters reside. Nevertheless, the key finding of this preliminary study is that cancer cells treated with an appropriately designed, inert metal-based probe can be readily visualized in noncanonical regions of the IR spectrum. This approach may pave the way for functional imaging of cells exhibiting a glycolytic phenotype.

## 3. Materials and Methods

### 3.1. Synthesis: General Procedures and Materials

All solvents and chemicals were used as purchased without further purification. Chromatographic separations were performed on silica gel columns by flash chromatography (Kieselgel 40, 0.040–0.063 mm; Merck, Darmstadt, Germany). Reactions were followed by thin layer chromatography (TLC) on Merck aluminum silica gel (60 F_254_) sheets that were visualized under a ultraviolet (UV) lamp. Evaporation was performed *in vacuo* (rotating evaporator). Sodium sulfate was always used as the drying agent. Proton (^1^H, 400 MHz) and carbon (^13^C, 100 MHz) NMR spectra were obtained with a Bruker Avance III 400 MHz spectrometer (Billerica, MA, USA) using the indicated deuterated solvents. Chemical shifts are given in parts per million (ppm) (δ relative to residual solvent peak for ^1^H and ^13^C). ^1^H-NMR spectra are reported in this order: multiplicity and number of protons. Standard abbreviations indicating the multiplicity were used as follows: s = singlet, d = doublet, dd = doublet of doublets, t = triplet, dt = doublet of triplets, m = multiplet. HPLC analysis was used to determine purity: all final compounds (i.e., assessed in biological assays) were ≥95% pure by HPLC, as confirmed via UV detection (λ = 254 nm). Analytical reversed-phase HPLC was conducted using a Kinetex EVO C18 column (5 μm, 150 × 4.6 mm, Phenomenex, Inc., Torrance, CA, USA); eluent A, water; eluent B, CH_3_CN; after 5 min. at 25% B, a gradient was formed from 25% to 75% of B in 5 min and held at 75% of B for 10 min; flow rate was 1 mL/min. HPLC analyses were performed at 254 nm. The ESI-MS spectra were recorded by direct injection at a 5 μL min^−1^ flow rate in an Q Exactive Plus Orbitrap high-resolution mass spectrometer (Thermo Fisher Scientific, Inc., Waltham, MA, USA), equipped with a HESI source. The working conditions were as follows: positive polarity, spray voltage 3.4 kV, capillary temperature 290 °C, S-lens RF level 50, the sheath gas was set at 24 and the auxiliary gas was set at 5 (arbitrary units), respectively. For acquisition and analysis, Xcalibur 4.2 software (Thermo Fisher Scientific, Inc.) was used. For spectra acquisition, a nominal resolution (at *m*/*z* 200) of 140,000 was used. IR spectra of final compounds were recorded on Cary 660 FTIR spectrometer (Agilent Technologies, Inc., Santa Clara, CA, USA) and data are reported as absorbance frequency (wavenumbers, cm^−1^). IR images were acquired using an Agilent Technologies FTIR 620 microscope equipped with a Focal Plane Array (FPA) detector cooled by liquid nitrogen. Yields refer to isolated and purified products derived from non-optimized procedures. Compounds **1**, 2-azidoethanol, **6** and **7** were synthesized as previously reported [16,17,19,21].

#### 3.1.1. Procedure for the Synthesis of Compound **2**

In a two-neck round-bottom flask, under inert atmosphere, 2,3,4,6-tetra-*O*-acetyl-α-*D*-glucopyranosyl trichloroacetimidate **1** [16] (0.240 mmol, 1.00 equiv) was dissolved in anhydrous toluene (1.0 mL). The solution was co-evaporated with toluene to remove residual water via azeotrope formation, and the resulting residue was further dried under high vacuum for 2 h. The residue was then dissolved in 5.0 mL of anhydrous CH_2_Cl_2_, and activated molecular sieves UOP Type 3Å (AW-300, Merck KGaA, Darmstadt, Germany), previously dried under vacuum for 2 h, were added to the solution. Subsequently, 2-azidoethanol [17], as a 1.3 M solution in CH_2_Cl_2_ (0.55 mL, 0.71 mmol, 3.0 equiv), was added. The reaction mixture was stirred at −20 °C under inert atmosphere. After 30 min, 0.5 mL of a 0.1 M solution of trimethylsilyl trifluoromethanesulfonate (TMSOTf) in anhydrous CH_2_Cl_2_ (0.0500 mmol of TMSOTf, 0.200 equiv) was added, and the reaction mixture was stirred at room temperature. After 24 h, TLC analysis showed the disappearance of the starting material. The reaction mixture was diluted with CH_2_Cl_2_, neutralized with Et_3_N, filtered through a Celite pad, and washed with a CH_2_Cl_2_/MeOH (95:5) solution. After solvent removal under reduced pressure, the crude product was purified by silica gel column chromatography using *n*-hexane/EtOAc (6:4) as the eluent.

*(2R*,*3R*,*4S*,*5R*,*6R)-2-(Acetoxymethyl)-6-(2-azidoethoxy)tetrahydro-2H-pyran-3*,*4*,*5-triyl triacetate* (**2**). White solid. 45% yield from **1**. ^1^H NMR (CDCl_3_) δ (ppm): 1.98 (s, 3H), 2.01 (s, 3H), 2.03 (s, 3H), 2.07 (s, 3H), 3.23–3.29 (m, 1H), 3.45–3.51 (m, 1H), 3.64–3.71 (m, 2H), 3.99–4.04 (m, 1H), 4.16 (dd, 1H, *J* = 12.0, 2.4 Hz), 4.25 (dd, 1H, *J* = 12.0, 4.4 Hz), 4.58 (d, 1H, *J* = 8.0 Hz), 5.03 (dd, 1H, *J* = 9.6, 8.0 Hz), 5.10 (t, 1H, *J* = 9.6 Hz), 5.21 (t, 1H, *J* = 9.6 Hz).

#### 3.1.2. Procedure for the Synthesis of Compound **4**

In a flame-dried two-necked round bottomed flask, the iron catalyst [Fe_2_(CO)_9_] (2.5 mol %) was added under argon atmosphere. Then commercially available pinacolborane (1.25 equiv), commercially available 1,7-octadiyne **3** (9.42 mmol, 1.00 equiv) and dry toluene (9.6 mL) were added. The resulting dark mixture was refluxed at 100 °C 16 h. After consumption of the starting material (TLC), the reaction mixture was cooled down to room temperature under argon flux and the solvent was evaporated under reduced pressure. The crude product was purified by flash chromatography on silica gel (eluent mixture *n*-hexane/Et_2_O 10:0.8).

*(E)-4*,*4*,*5*,*5-Tetramethyl-2-(pent-1-en-4-yn-1-yl)-1*,*3*,*2-dioxaborolane* (**4**). Yellow oil. 32% yield from **3**. ^1^H NMR (CDCl_3_) δ (ppm): 1.26 (s, 12H), 1.50–1.58 (m, 4H), 1.93 (t, 1H, *J* = 2.6 Hz), 2.13–2.22 (m, 4H), 5.44 (dt, 1H, *J* = 17.9, 1.6 Hz), 6.62 (dt, 1H, *J* = 17.9, 6.4 Hz).

#### 3.1.3. Procedure for the Synthesis of Compound **5**

In a one-necked round bottomed flask, intermediate **4** (1.79 mmol, 1.00 equiv) was dissolved in acetone (18.4 mL). Then NaIO_4_ (3.03 equiv), NH_4_OAc (2.23 equiv) and distilled H_2_O (18.4 mL) were added at room temperature. The reaction mixture was stirred at room temperature 16 h. After consumption of the starting material (TLC), acetone was quickly evaporated under reduced pressure. The resulting mixture was extracted with distilled H_2_O and EtOAc (x2) and the organic phase was washed with an aqueous solution of 1N HCl and subsequently dried over sodium sulfate, filtered and concentrated under reduced pressure. The desired compound obtained was used in the next step without further purification.

*(E)-Pent-1-en-4-yn-1-ylboronic acid* (**5**). Yellow solid. 92% yield from **4**. ^1^H NMR (CDCl_3_) δ (ppm): 1.51–1.64 (m, 4H), 1.95 (t, 1H, *J* = 2.6 Hz), 2.16–2.28 (m, 4H), 5.55 (d, 1H, *J* = 17.6 Hz), 6.95 (dt, 1H, *J* = 17.7, 6.5 Hz).

#### 3.1.4. Procedure for the Synthesis of Compound **8**

[Re(CO)_3_(CH_3_CN)_3_]OTf **6** [21] (1.00 equiv) was added under argon atmosphere in a flame-dried two-necked round bottomed flask containing the solution of boronic acid 5 (2.08 mmol, 2.00 equiv) in anhydrous CH_3_CN (20.8 mL). Thereafter, phosphazine **7** [19] (3.0 mmolP/g, 80% functionalized, 1.50 equiv) and Et_3_N (4.00 equiv) were added. The mixture was stirred at 80 °C 16 h. The day after, the reaction mixture was cooled down to room temperature under argon atmosphere and then filtered on a Celite pad. The solution obtained was concentrated under *vacuum* and purified by flash column chromatography (eluent mixture *n*-hexane/Et_2_O 10:0.7) to obtain the desired compound.

*1-(Oct-1-en-7-ynyl)-cyclopentadienyl tricarbonyl rhenium* (**8**). Yellow oil. 14% yield from **5** and **6**. ^1^H NMR (CDCl_3_) δ (ppm): 1.59–1.57 (m, 4H), 1.95 (t, 1H, *J* = 2.7 Hz), 2.12–2.23 (m, 4H), 5.27 (*pseudo* t, 2H, *J* = 2.2 Hz), 5.41 (*pseudo* t, 2H, *J* = 2.2 Hz), 5.94–5.97 (m, 2H).

#### 3.1.5. Procedure for the Synthesis of Compound **9**

Alkyne **8** (0.104 mmol, 1.00 equiv) and azide **2** (1.00 equiv) were suspended in a 1:1 *v*/*v* mixture of 1,4-dioxane and *tert*-butyl alcohol (3.5 mL). A freshly prepared sodium ascorbate aqueous solution (1.00 equiv, 1.8 mL of water) was added, followed by copper (II) sulfate pentahydrate aqueous solution (0.500 equiv, 1.8 mL of water). The heterogeneous mixture was stirred vigorously at 100 °C in a sealed vial. After the consumption of the starting material (TLC), the reaction mixture was cooled and filtered on a Celite pad. The crude product was purified by flash column chromatography (eluent mixture *n*-hexane/ethyl acetate 4:6) to obtain the desired compound.

*((E)-6-(1-(2-(1-(2*,*3*,*4*,*6-tetra-O-Acetyl-β-D-glucopyranoside))ethyl)-1H-1*,*2*,*3-triazol-4-yl)hex-1-en-1-yl)cyclopentadienyl tricarbonyl rhenium* (**9**). Off-white solid. 80% Yield from 2 and 8. ^1^H NMR (CDCl_3_) δ (ppm): 1.44–1.53 (m, 2H), 1.65–1.74 (m, 2H), 1.95 (s, 3H), 2.00 (s, 3H), 2.03 (s, 3H), 2.09 (s, 3H), 2.14–2.20 (m, 2H), 2.66–2.75 (m, 2H), 3.66–3.73 (m, 1H), 3.87–3.96 (m, 1H), 4.09–4.16 (m, 2H), 4.18–4.28 (m, 2H), 4.47 (d, 1H, *J* = 8.0 Hz), 4.53–4.61 (m, 1H), 4.99 (dd, 1H, *J* = 9.6, 8.0 Hz), 5.04–5.10 (m, 1H), 5.15–5.21 (m, 1H), 5.27 (*pseudo* t, 2H, *J* = 2.2 Hz), 5.42 (*pseudo* t, 2H, *J* = 2.2 Hz), 5.94–5.98 (m, 2H), 7.35 (s, 1H). ^13^C-NMR (CDCl_3_) δ (ppm): 20.71 (4C), 20.77, 20.88, 28.71 (2C), 32.29 (2C), 61.90, 68.36, 71.12, 72.14, 72.63 (2C), 81.02, 84.03 (2C), 84.04 (2C), 100.70, 107.18, 120.58, 133.52, 169.41, 169.56, 170.25, 170.72, 194.52 (3C).

#### 3.1.6. Procedure for the Synthesis of Compound **10**

In a flame-dried two-necked round bottomed flask under argon atmosphere, compound **9** (0.0582 mmol, 1.00 equiv) was dissolved in anhydrous MeOH (2.0 mL). The solution was cooled down to 0 °C and MeONa (s) (37.7 mg, 12.0 eq) was then added. The mixture was left under stirring at room temperature for 51 h. After the consumption of the starting material (TLC), the mixture was diluted with MeOH and filtered on a Celite pad. The crude product was purified by flash column chromatography (eluent mixture CHCl_3_/MeOH 9:1).

*((E)-6-(1-(2-(1-β-D-Glucopyranoside)ethyl)-1H-1*,*2*,*3-triazol-4-yl)hex-1-en-1-yl) cyclopentadienyl tricarbonyl rhenium* (**10**). White solid. 83% yield from **9**. ^1^H NMR (CD_3_OD) δ (ppm): 1.47 (quint, 2H, *J* = 7.5 Hz, CH_2_), 1.69 (quint, 2H, *J* = 7.6 Hz, CH_2_), 2.14–2.22 (m, 2H, CH_2_), 2.70 (t, 2H, *J* = 7.5 Hz, CH_2_), 3.18 (dd, 1H, *J* = 9.1, 7.8 Hz, Glc-H), 3.24–3.29 (m, 2H, 2×Glc-H), 3.32–3.37 (m, 1H, Glc-H), 3.61–3.69 (m, 1H, Glc-H), 3.86 (dd, 1H, *J* = 11.9, 1.5 Hz, Glc-H), 3.98 (dt, 1H, *J* = 11.7, 5.2 Hz, one of the two diastereotopic Alk-CH_2_-O-Glc), 4.23 (dt, 1H, *J* = 11.7, 4.9 Hz, one of the two diastereotopic Alk-CH_2_-O-Glc), 4.30 (d, 1H, *J* = 7.8 Hz, anomeric Glc-H1), 4.60 (t, 2H, *J* = 5.1 Hz, CH_2_-triazole), 5.43 (*pseudo* t, 2H, *J* = 2.2 Hz, Cp), 5.66 (*pseudo* t, 2H, *J* = 2.2 Hz, Cp), 5.99–6.13 (m, 2H, -HC=CH-), 7.86 (s, 1H, H-triazole). ^13^C NMR (CD_3_OD) δ (ppm): 26.06 (CH_2_), 29.62 (CH_2_), 29.90 (CH_2_), 33.16 (CH_2_), 51.50 (Alk-CH_2_-triazole), 62.73 (Alk-CH_2_-O-Glc), 69.13 (Glc), 71.56 (Glc), 74.97 (Glc), 77.97 (Glc), 78.09 (Glc), 82.41 (2×Cp-CH), 85.32 (2×Cp-CH), 104.59 58 (anomeric Glc-C1), 108.57 (Cp-C), 121.91 (vinylic=CH), 124.27 (CH-triazole), 134.27 (vinylic=CH), 148.89 (C-triazole), 195.90 (3×CO). FTIR: $\bar{\nu }$ 1897, 2007, 2851, 2919, 3386 cm^−1^. HPLC analysis: retention time = 10.773 min; peak area, 96% (254 nm). HRMS: *m*/*z* for C_24_H_30_N_3_O_9_ReNa [M + Na]^+^ calculated: 714.14373, found: 714.14240.

#### 3.1.7. Procedure for Compound **11**

In a one-necked round bottomed flask, compound **10** (0.0448 mmol, 1.00 equiv) was solubilized in HPLC MeOH (2.2 mL) and added of 10% Pd/C (12.5 mg for 0.0448 mmol of starting material). The reaction was stirred under H_2_ atmosphere at room temperature 16 h. After the consumption of the starting material (TLC), the reaction mixture was filtered on a Celite pad and washed with HPLC-grade MeOH. The solvent was evaporated under reduced pressure, and the residue was dried under *vacuum* to give pure compound **11** without any further purification.

*(6-(1-(2-(1-β-D-Glucopyranoside)ethyl)-1H-1*,*2*,*3-triazol-4-yl)hexyl)cyclopentadienyl tricarbonyl rhenium* (**11**). White solid. 79% yield from **10**. ^1^H NMR (CD_3_OD) δ (ppm): 1.36–1.46 (m, 4H, 2×CH_2_), 1.49–1.59 (m, 2H, CH_2_), 1.63–1.73 (m, 2H, CH_2_), 2.43 (*pseudo* t, 2H, *J* = 7.7 Hz, CH_2_), 2.69 (t, 2H, *J* = 7.6 Hz, CH_2_), 3.17 (dd, 1H, *J* = 9.0, 7.7 Hz, Glc-H), 3.24–3.29 (m, 2H, 2×Glc-H), 3.32–3.38 (m, 1H, Glc-H), 3.62–3.68 (m, 1H, Glc-H, 3.87 (dd, 1H, *J* = 11.8, 1.6 Hz, Glc-H), 3.98 (dt, 1H, *J* = 11.4, 5.3 Hz, one of the two diastereotopic Alk-CH_2_-O-Glc), 4.23 (dt, 1H, *J* = 11.7, 5.1 Hz, one of the two diastereotopic Alk-CH_2_-O-Glc), 4.30 (d, 1H, *J* = 7.8 Hz, anomeric Glc-H1), 4.60 (t, 2H, *J* = 5.1 Hz, CH_2_-triazole), 5.39 (*pseudo* t, 2H, *J* = 2.1 Hz. Cp), 5.44 (*pseudo* t, 2H, *J* = 2.1 Hz, Cp), 7.86 (s, 1H, H-triazole). ^13^C NMR (CD_3_OD) δ (ppm): 26.21 (CH_2_), 29.10 (CH_2_), 29.86 (CH_2_), 30.04 (CH_2_), 30.39 (CH_2_), 32.78 (CH_2_), 51.50 (Alk-CH_2_-triazole), 62.72 (Alk-CH_2_-O-Glc), 69.14 (Glc), 71.57 (Glc), 74.97 (Glc), 77.97 (Glc), 78.09 (Glc), 84.54 (2×Cp-CH), 84.90 (2×Cp-CH), 104.58 (anomeric Glc-C1), 113.27 (Cp-C), 124.24 (CH-triazole), 149.01 (C-triazole), 196.08 (3×CO). FTIR: $\bar{\nu }$ 1907, 2016, 2857, 2928, 3338, 3368 cm^−1^. HPLC analysis: retention time = 10.979 min; peak area = 95% (254 nm). HRMS: *m*/*z* for C_24_H_32_N_3_O_9_ReNa [M + Na]^+^ calculated: 716.15938, found: 716.15826.

### 3.2. In Vitro FTIR Microscope Imaging and Detection of Portions (Cells/Tissues) of Pancreatic Adenocarcinoma (PDAC)

Primary cell cultures of pancreatic tumor (PCC-PDAC) were cultured in supplemented RPMI medium, at 37 °C and 5% CO_2_ as reported previously [22]. GLUT1 expression levels were quantified by quantitative PCR using a TaqMan Gene Expression Assay (Hs01102423_m1, Thermo Fisher Scientific, Waltham, MA, USA), following protocols described by Sciarrillo et al. [23]. These cells were placed on Kevley Technologies trans-reflectance MirrIR slides. In particular, chamber-slides were assembled on the MirrIR slides, where PCC-PDAC cells could be cultured and treated with the IR probe (100 µM) for 1 h, then rinsed and replaced by fresh medium. The biological material used in the experiments was afterward stored at −80 °C. The slides obtained were analyzed by means of an Agilent Technologies FTIR 620 microscope equipped with a Focal Plane Array (FPA) detector cooled by liquid nitrogen. Each analysis was carried out in reflectance modality with 64 scans, both for the samples and for background, at a resolution of 4 cm^−1^ in a spectral range of 3500 to 900 cm^−1^. For each analyzed spectrum, both the IR image in fake colors (FPA images) and the visible image, measuring a size of 350 × 350 µm each, were recorded.

### 3.3. Evaluation of Inhibition of Cell Growth Using the Sulforhodamine B (SRB) Assay

Cellular sensitivity to compound **11** was measured with the sulforhodamine B (SRB) assay. Primary cell cultures of pancreatic tumor (PCC-PDAC) were cultured in supplemented RPMI medium, at 37 °C and 5% CO_2_ as reported previously [22]. PDAC cells (5000 cells per well) were seeded in 96-well plates and exposed to the compound for 1 and 72 h, and inhibition of cell growth was determined as described previously [22]. Cells were seeded in triplicate into 96-well flat-bottom plates at a density in a total volume of 100 µL per well and incubated at 37 °C for 24 h to allow formation of a confluent monolayer. After incubation, 100 µL of the test compound, dissolved in DMSO at varying concentrations, were added to each well. The cells were then incubated for 1 or 72 h under controlled conditions (37 °C, 5% CO_2_, and 100% humidity). At the end of the treatment period, the cells were fixed by adding 25 µL of 50% cold trichloroacetic acid and incubated at 4 °C for at least 60 min. The plates were then emptied, gently washed with deionized water, and air-dried overnight at room temperature (RT). The cells were subsequently stained with 50 µL of 0.4% SRB solution prepared in 1% acetic acid for 15 min at RT. Excess SRB was removed by gently washing the plates with 1% acetic acid, followed by air-drying overnight at RT. The protein-bound SRB dye was then solubilized with 150 µL of TRIS buffer solution (pH 8.8). Plates were placed on a microplate shaker for four minutes prior to reading. Absorbance was measured at 490 nm using a microplate reader. Cell growth inhibition was calculated as the percentage of drug-treated cells relative to vehicle-treated control cells (untreated cells) (Figure S10).

## 4. Conclusions

Application of FTIR spectroscopy to functional imaging still is a relatively unexplored diagnostic technique, in spite of being relatively economical, simple, fast, and non-dangerous. Although IR signals penetrate cellular layers less effectively than radionuclide radiation, there remains substantial scope to improve this technique. We have developed the first example of a glucoconjugated organometallic cyclopentadienyl *fac*-[Re(CO)_3_] complex (compound **11**), based on a site-specific functionalization of a glucose unit. This complex can serve as an IR probe, enabling the visualization of cancer cells at an interference-free spectral frequency by exploiting their elevated uptake of glucose. Future efforts will be dedicated to establishing the extent of the selectivity of these probes in cancer cells, as compared with their healthy counterparts.

## Data Availability

The original contributions presented in this study are included in the article/Supplementary Material. Further inquiries can be directed to the corresponding author.

## Supplementary Materials

The following supporting information can be downloaded at: https://www.mdpi.com/article/10.3390/molecules31010028/s1, Figure S1: RP-HPLC trace of final compound; Figure S2: ^1^H-NMR (CD_3_OD, 400 MHz) of compound **11**. Figure S3: ^13^C-NMR (CD_3_OD, 100 MHz) of compound **11**. Figure S4: ESI-HRMS spectrum of final compound. Figure S5: mRNA expression levels of GLUT1 in PDAC cells compared to HPDE cells. Figure S6: HPLC chromatogram of complex **11** (250 μM) in buffered medium at pH 7.3 immediately after preparation (t1 = 0 min). Figure S7: HPLC chromatogram of complex **11** (250 μM) in buffered medium at pH 7.3 after 2 h (t2 = 2 h). Figure S8: HPLC chromatogram of complex **11** (250 μM) in buffered medium at pH 7.3 after 24 h (t3 = 24 h). Figure S9: 2D IR absorption intensity of PDAC cells treated with unsubstituted Cp[Re(CO)_3_] complex at 2100 cm^−1^. Figure S10: Cytotoxicity evaluation of compound **11** in PDAC cells at 1 h and 72 h. Figure S11: ^1^H-NMR (CDCl_3_, 400 MHz) of intermediate **9**. Figure S12: ^13^C-NMR (CDCl_3_, 100 MHz) of intermediate **9**. Figure S13: ^1^H-NMR (CD_3_OD, 400 MHz) of intermediate **10**. Figure S14: ^13^C-NMR (CD_3_OD, 100 MHz) of intermediate **10**.

## Abbreviations

The following abbreviations are used in this manuscript:

ATP	Adenosine Triphosphate
Cp[Re(CO)_3_]	Cyclopentadienylrhenium(I) Tricarbonyl
FDG	Fluorodeoxyglucose
FPA	Focal Panel Array
FTIR	Fourier Transform Infrared Spectroscopy
GLUT1	Glucose Transporter 1
HPDE	Human Pancreatic Duct Epithelial Cells
HPLC	High Performance Liquid Chromatography
HRMS	High-Resolution Mass Spectrometry
IR	Infrared
NMR	Nuclear Magnetic Resonance
PCC-PDAC	Primary Cell Cultures of Pancreatic Ductal Adenocarcinoma
PCR	Polymerase Chain Reaction
PDAC	Pancreatic Ductal Adenocarcinoma
PET	Positron Emission Tomography
ppm	parts per million
RT	Room Temperature
TLC	Thin Layer Chromatography
UV	Ultraviolet
