# Supplementary material for: Synthesis and Application of a Glucoconjugated Organometallic Rhenium Complex as an IR Imaging Probe for Glycolytic Cancer Cells
Source: Molecules. 2025 Dec 22;31(1):28. doi: 10.3390/molecules31010028 (PMC12786666; doi:10.3390/molecules31010028)
Supplement: Supplementary file 1 [file molecules-31-00028-s001.zip › molecules-4039423-supplementary.pdf]

Supporting Information

Synthesis and Application of a Glucoconjugated Organometallic Rhenium Complex as an IR Imaging Probe for Glycolytic Cancer Cells

Giulia Bononi [1,2], Erica Paltrinieri [1], Serena Fortunato [1,†], Gaspare Cicio [1,‡], Nicola Di Giovanni [1,§], Giulia Lencioni [3], Niccola Funel [4], Elisa Giovannetti [3,5], Carlotta Granchi [1,2], Valeria Di Bussolo [1] and Filippo Minutolo [1,2,*]

[1] Department of Pharmacy, University of Pisa, Via Bonanno 6, 56126 Pisa, Italy; giulia.bononi@unipi.it (G.B.); serena.fortunato@unipi.it (S.F.); carlotta.granchi@unipi.it (C.G.); valeria.dibussolo@unipi.it (V.D.B.)
[2] Center for Instrument Sharing of the University of Pisa (CISUP), Lungarno Pacinotti 43, 56126 Pisa, Italy
[3] Cancer Pharmacology Laboratory, AIRC Start-Up Unit, Fondazione Pisana per la Scienza, Via Ferruccio Giovannini, 13, San Giuliano Terme, 56017 Pisa, Italy; g.lencioni@fpscience.it (G.L.)
[4] USL Tuscany Northwest Location Lucca, Department of Laboratory Diagnostics, Via Lippi Francesconi, 55100 Lucca, Italy; niccola.funel@uslnordovest.toscana.it
[5] Department of Medical Oncology, Cancer Center Amsterdam, Amsterdam University Medical Center, Vrije Universiteit Amsterdam, 1081 HV Amsterdam, The Netherlands
[*] Correspondence: filippo.minutolo@unipi.it
[†] Current address: Department of Research and Knowledge Enhancement, University of Pisa, Lungarno Pacinotti 43/44, 56126 Pisa, Italy.
[‡] Current address: Menarini Ricerche SpA, Via Livornese 897, 56122 Pisa, Italy.
[§] Current address: Menarini Ricerche SpA, Via dei Sette Santi 1, 50131 Firenze, Italy.

Table of Contents

Figure S1. RP-HPLC trace of final compound. S3

Figure S2. ^{1}H-NMR (CD_3OD, 400 MHz) of compound **11**. S4

Figure S3. ^{13}C-NMR (CD_3OD, 100 MHz) of compound **11**. S5

Figure S4. ESI-HRMS spectrum of final compound. S6

Figure S5. mRNA expression levels of GLUT1 in PDAC cells compared to HPDE cells. S7

Figure S6. HPLC chromatogram of complex **11** (250 μM) in buffered medium at pH 7.3 immediately after preparation (t1 = 0 min). S8

Figure S7. HPLC chromatogram of complex **11** (250 μM) in buffered medium at pH 7.3 after 2 hours (t2 = 2 h). S9

Figure S8. HPLC chromatogram of complex **11** (250 μM) in buffered medium at pH 7.3 after 24 hours (t3 = 24 h). S10

Figure S9. 2D IR absorption intensitiy of PDAC cells treated with unsubstituted Cp[Re$(CO)_3$] complex at 2100 cm^{-1}. S11

Figure S10. Cytotoxicity evaluation of compound **11** in PDAC cells at 1 h and 72 h. S11

Figure S11. ^{1}H-NMR ($CDCl_3$, 400 MHz) of intermediate **9**. S12

Figure S12. ^{13}C-NMR ($CDCl_3$, 100 MHz) of intermediate **9**. S13

Figure S13. ^{1}H-NMR (CD_3OD, 400 MHz) of intermediate **10**. S14

Figure S14. ^{13}C-NMR (CD_3OD, 100 MHz) of intermediate **10**. S15

==== Shimadzu LabSolutions Analysis Report ====

Sample Name : SF165_500uM
Sample ID : SF165_500uM
Data Filename : SF165_500uM_p_c.lcd
Method Filename : MAGL254.lcm

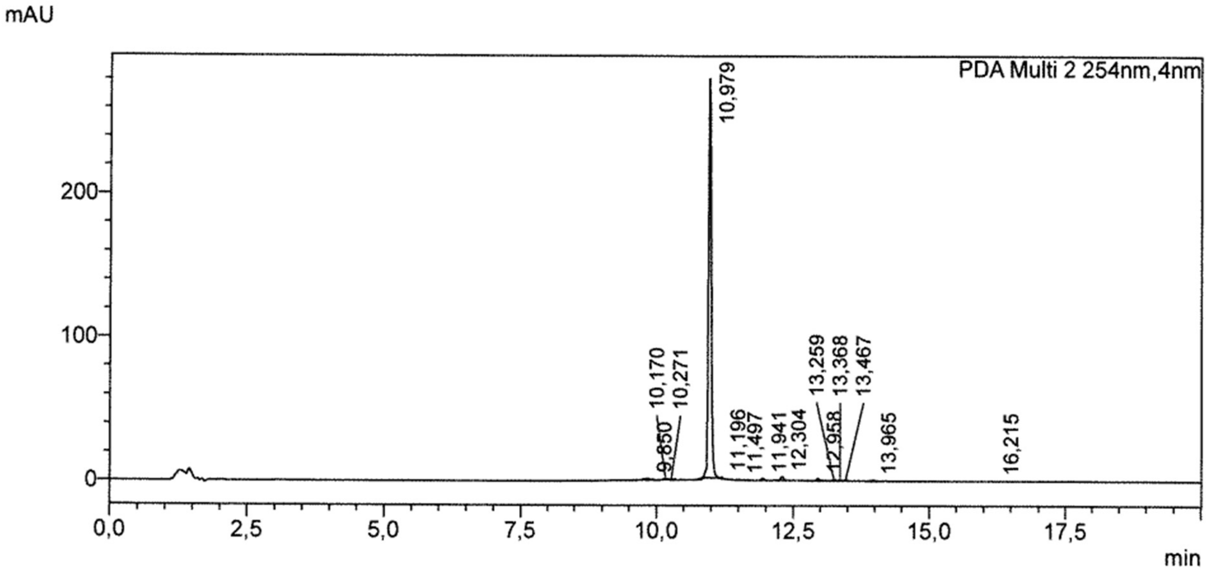

PDA Ch2 254nm

Peak#	Ret. Time	Area	Area%	Height
1	9,850	9696	0,947	1087
2	10,170	2911	0,284	661
3	10,271	1223	0,119	256
4	10,979	969193	94,611	277946
5	11,196	2904	0,283	1050
6	11,497	1339	0,131	288
7	11,941	5259	0,513	1319
8	12,304	12624	1,232	2705
9	12,958	7292	0,712	1503
10	13,259	3996	0,390	815
11	13,368	1717	0,168	376
12	13,467	2080	0,203	381
13	13,965	2942	0,287	594
14	16,215	1225	0,120	172
Total		1024402	100,000	289152

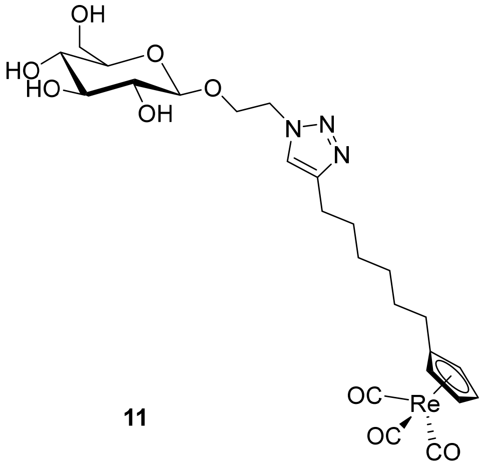

11

Figure S1. HPLC chromatogram of compound **11**.

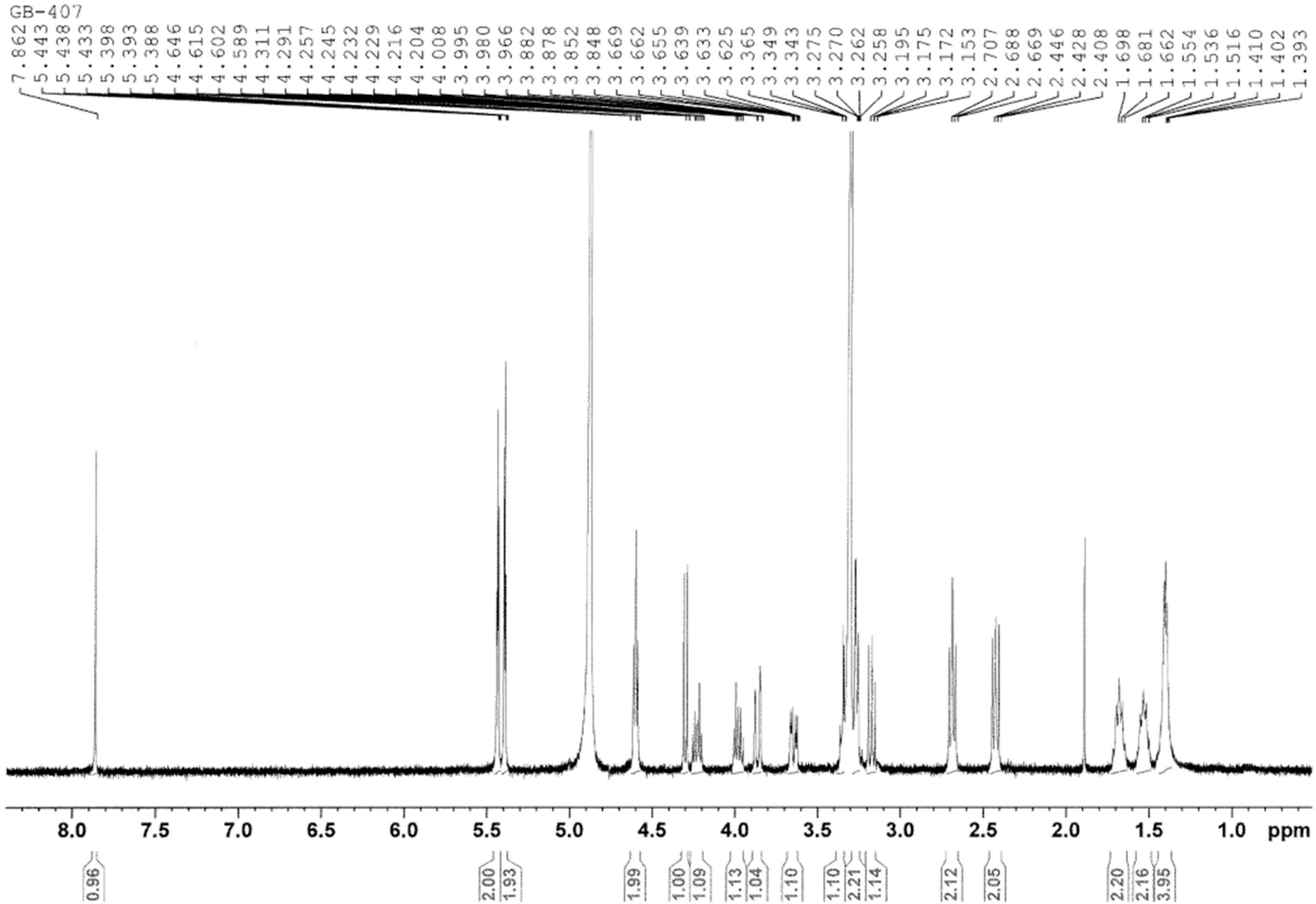

Figure S2. ^{1}H-NMR (CD_3OD, 400 MHz) of compound **11**.

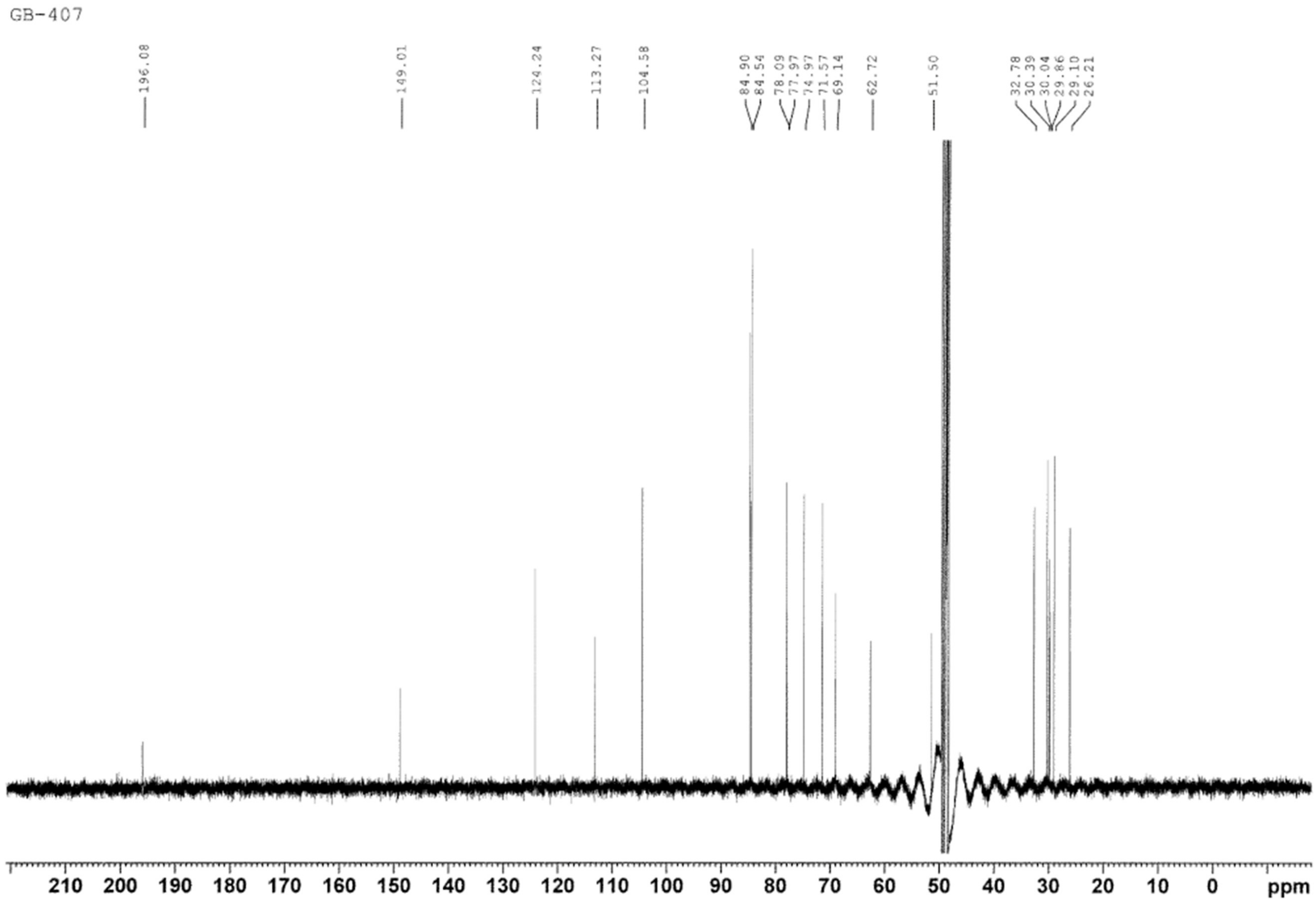

Figure S3. ^{13}C-NMR (CD_3OD, 100 MHz) of compound **11**.

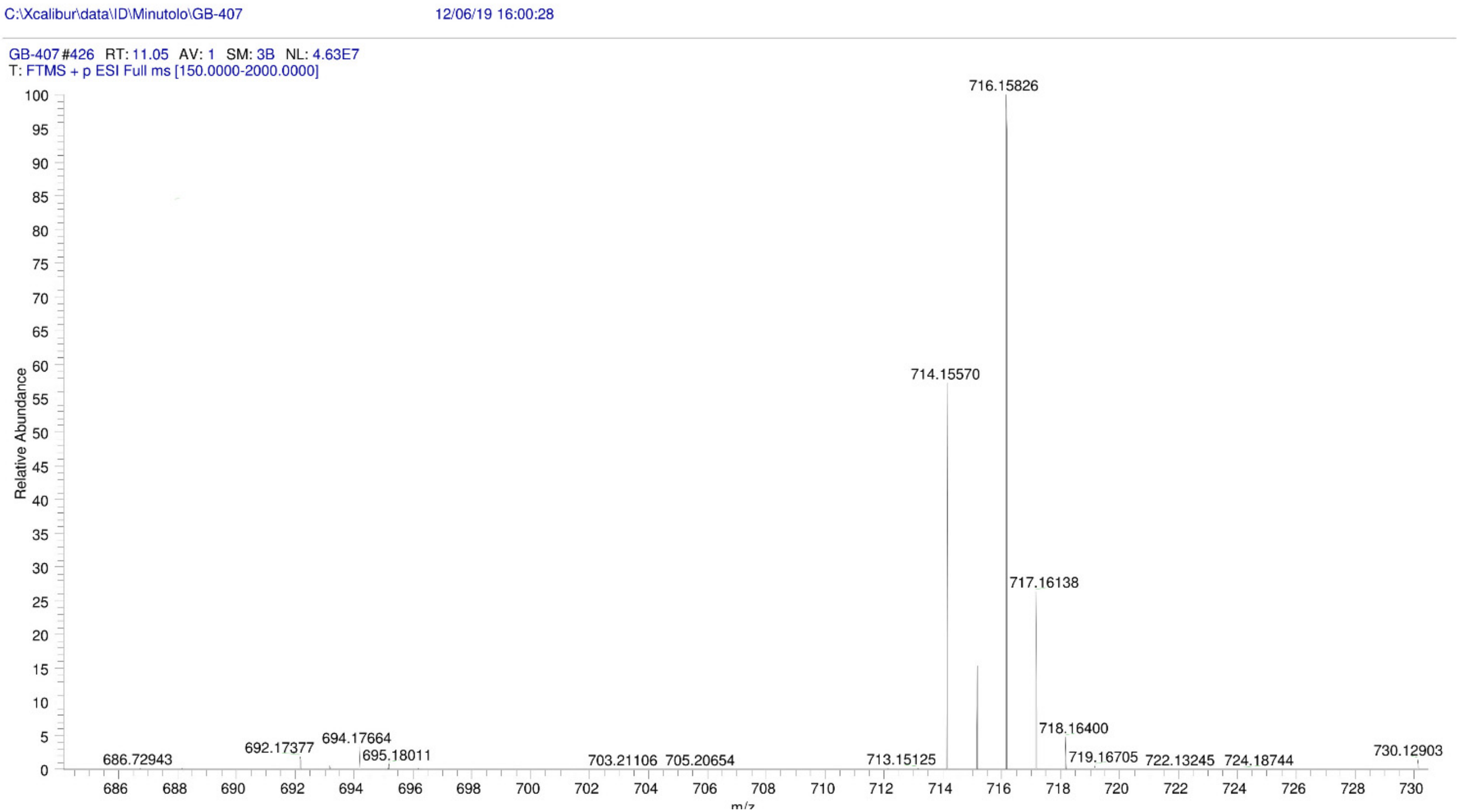

Figure S4. ESI-HRMS spectrum of compound **11**.

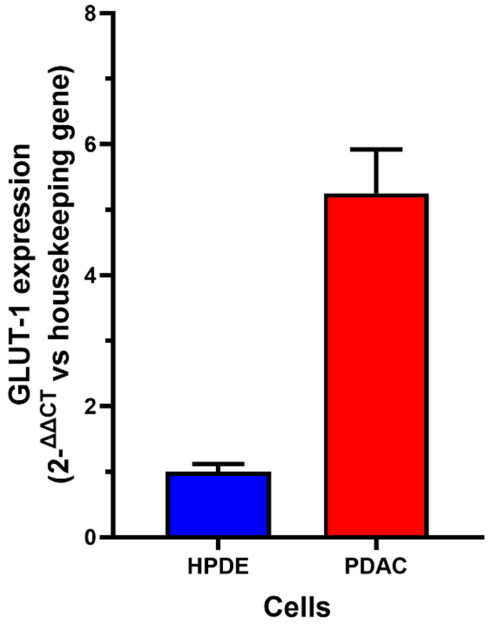

Figure S5. mRNA expression levels of GLUT1 in PDAC cells compared to HPDE cells.

==== Shimadzu LabSolutions Analysis Report ====

Sample Name : GB407_500uM_H2O_t1
Sample ID : GB407_500uM_H2O_t1
Data Filename : GB407_250uM_H2O_t1_p_c.lcd
Method Filename : MAGL254.lcm

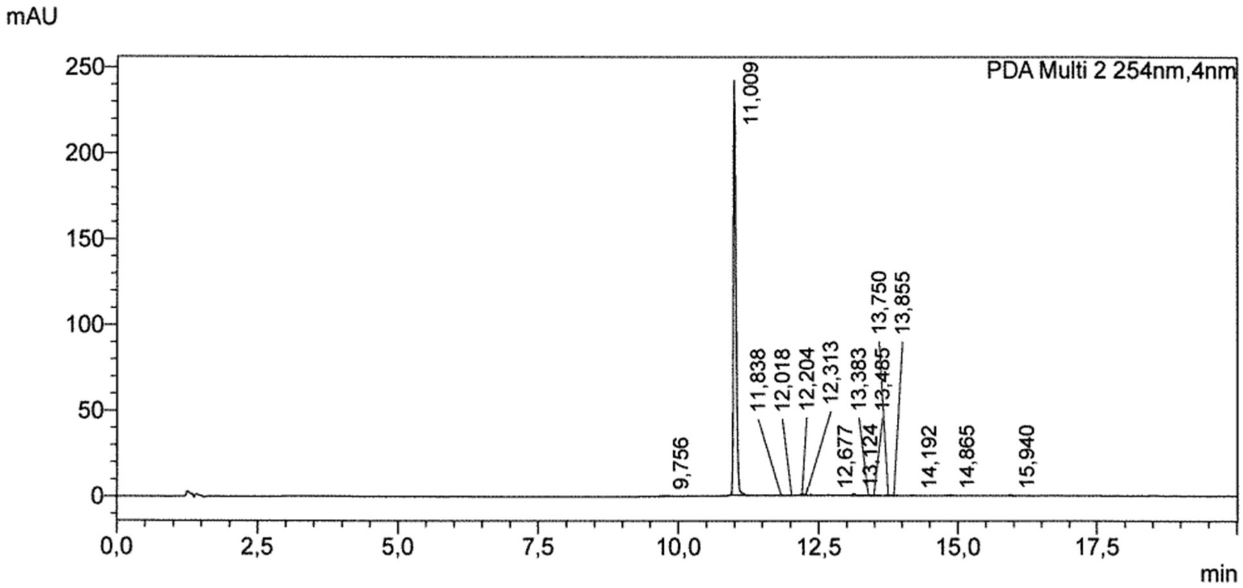

PDA Ch2 254nm

Peak#	Ret. Time	Area	Area%	Height
1	9,756	3235	0,399	481
2	11,009	766192	94,615	241614
3	11,838	1463	0,181	279
4	12,018	1027	0,127	159
5	12,204	3518	0,434	961
6	12,313	15816	1,953	4645
7	12,677	1184	0,146	339
8	13,124	4899	0,605	1098
9	13,383	1616	0,200	233
10	13,485	1989	0,246	232
11	13,750	2295	0,283	545
12	13,855	1399	0,173	228
13	14,192	1299	0,160	216
14	14,865	1555	0,192	282
15	15,940	2313	0,286	429
Total		809797	100,000	251741

Figure S6. HPLC chromatogram of complex **11** (250 μM) in buffered medium at pH 7.3 immediately after preparation (t1 = 0 min).

==== Shimadzu LabSolutions Analysis Report ====

Sample Name : GB407_250uM_H2O_t2
Sample ID : GB407_250uM_H2O_t2
Data Filename : GB407_250uM_H2O_t2_p_c.lcd
Method Filename : MAGL254.lcm

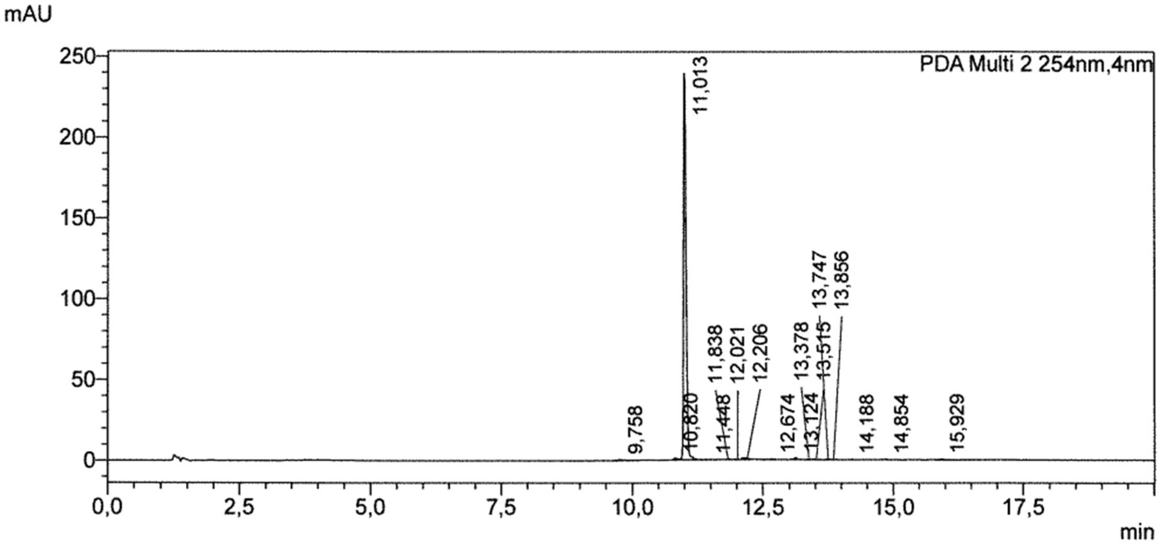

PDA Ch2 254nm

Peak#	Ret. Time	Area	Area%	Height
1	9,758	4181	0,508	517
2	10,820	5853	0,711	1118
3	11,013	784925	95,324	239100
4	11,448	3144	0,382	235
5	11,838	1881	0,228	356
6	12,021	1105	0,134	164
7	12,206	2101	0,255	693
8	12,674	1211	0,147	353
9	13,124	4694	0,570	1107
10	13,378	1703	0,207	238
11	13,515	1889	0,229	223
12	13,747	2426	0,295	554
13	13,856	1567	0,190	243
14	14,188	1479	0,180	247
15	14,854	2786	0,338	362
16	15,929	2484	0,302	452
Total		823428	100,000	245962

Figure S7. HPLC chromatogram of complex **11** (250 μM) in buffered medium at pH 7.3 after 2 hours (t2 = 2 h).

==== Shimadzu LabSolutions Analysis Report ====

Sample Name : GB407_250uM_H2O_t3
Sample ID : GB407_250uM_H2O_t3
Data Filename : GB407_250uM_H2O_t3_p_c.lcd
Method Filename : MAGL254.lcm

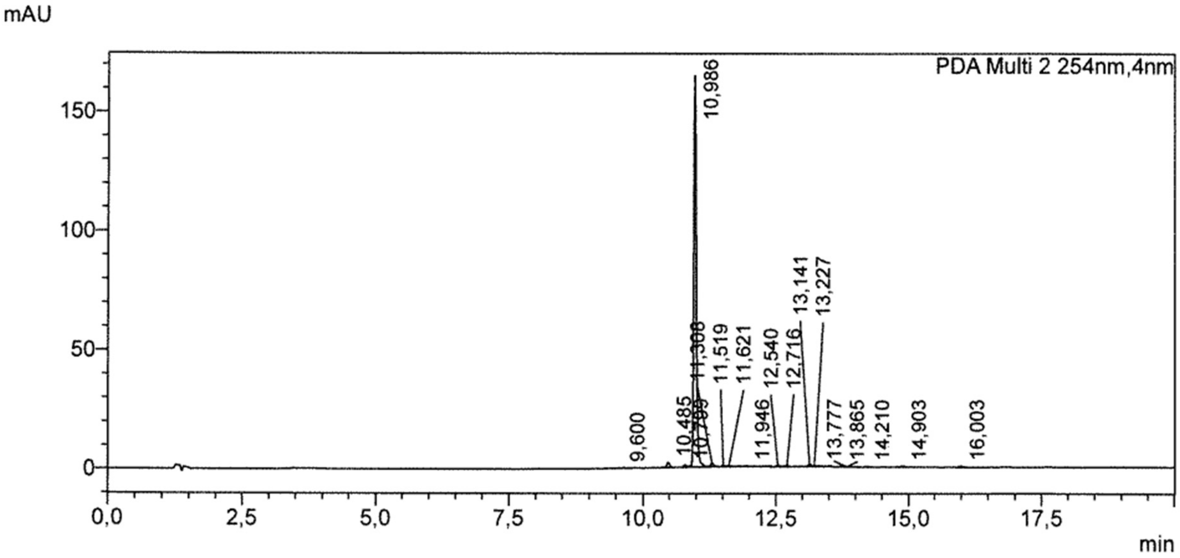

PDA Ch2 254nm

Peak#	Ret. Time	Area	Area%	Height
1	9,600	3287	0,572	163
2	10,485	7713	1,342	2049
3	10,799	4449	0,774	993
4	10,986	532491	92,679	164386
5	11,308	4984	0,867	1457
6	11,519	1282	0,223	398
7	11,621	1318	0,229	359
8	11,946	1019	0,177	248
9	12,540	1895	0,330	521
10	12,716	2680	0,466	518
11	13,141	2929	0,510	830
12	13,227	1118	0,195	262
13	13,777	2073	0,361	507
14	13,865	1859	0,324	331
15	14,210	1413	0,246	266
16	14,903	1731	0,301	323
17	16,003	2311	0,402	430
Total		574551	100,000	174041

Figure S8. HPLC chromatogram of complex **11** (250 µM) in buffered medium at pH 7.3 after 24 hours (t3 = 24 h).

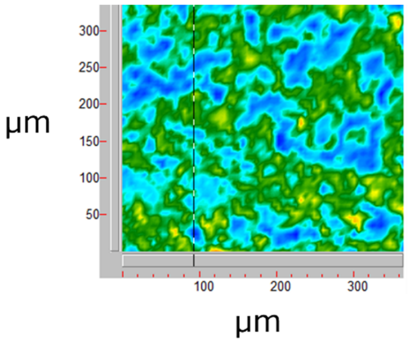

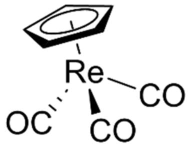

Figure S9. 2D IR absorption intensity of PDAC cells treated with unsubstituted Cp[Re(CO)3] complex at 2100 cm^{-1}.

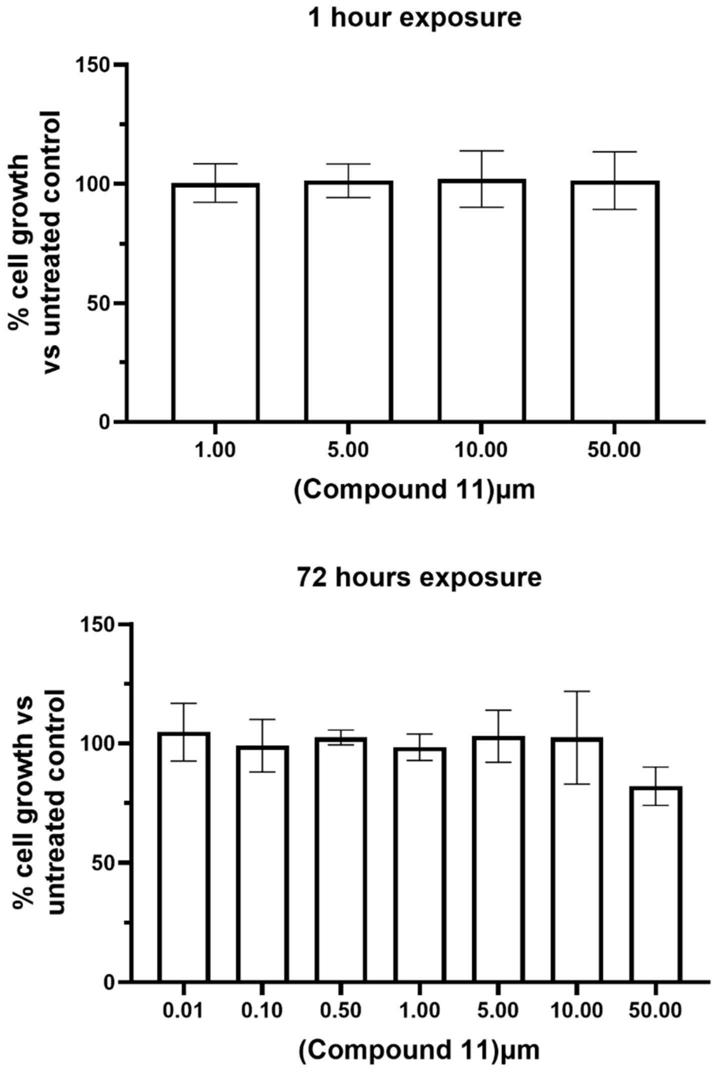

Figure S10. Cytotoxicity evaluation of compound **11** in PDAC cells at 1 h and 72 h.

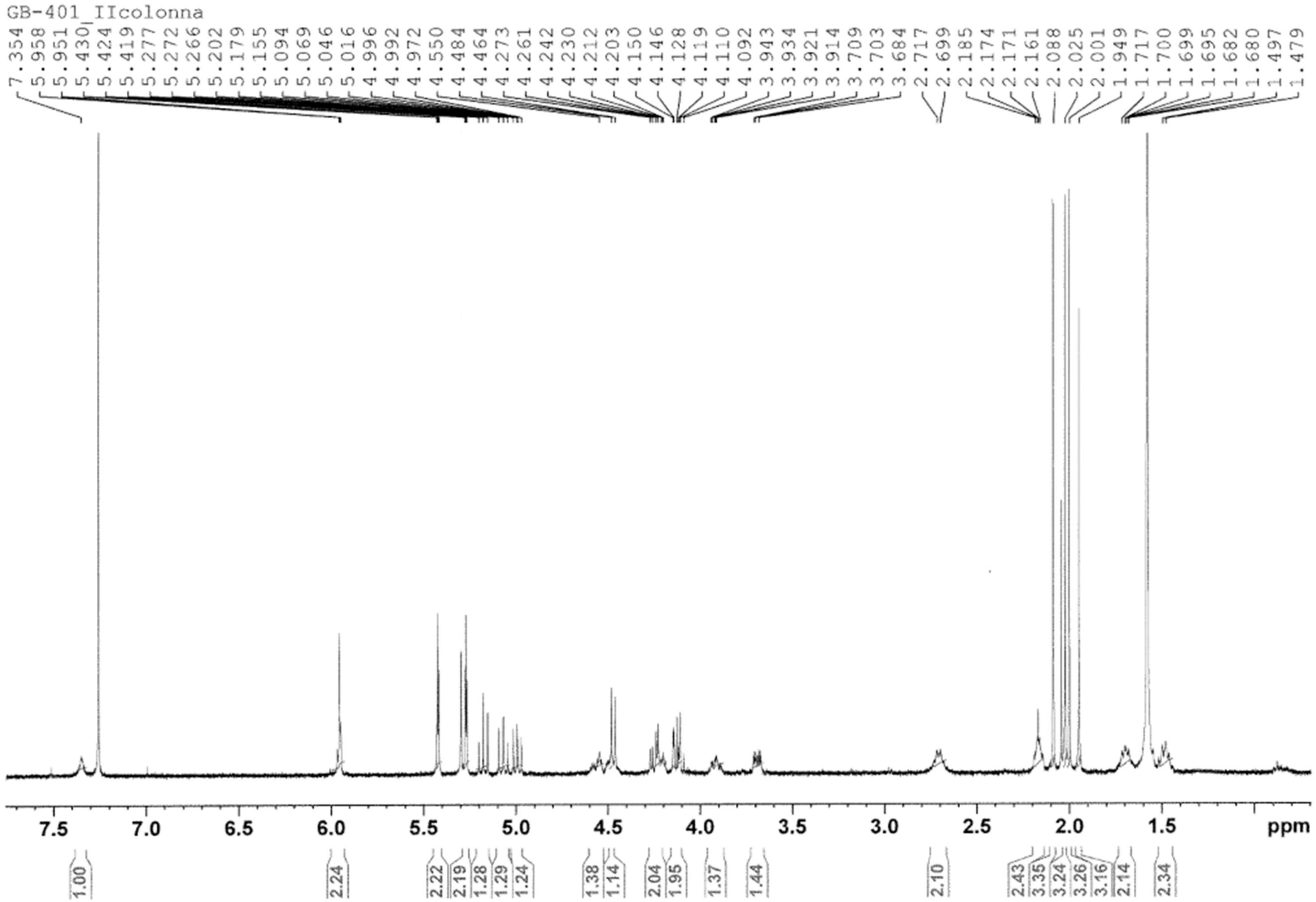

Figure S11. ^{1}H-NMR ($CDCl_3$, 400 MHz) of intermediate **9**.

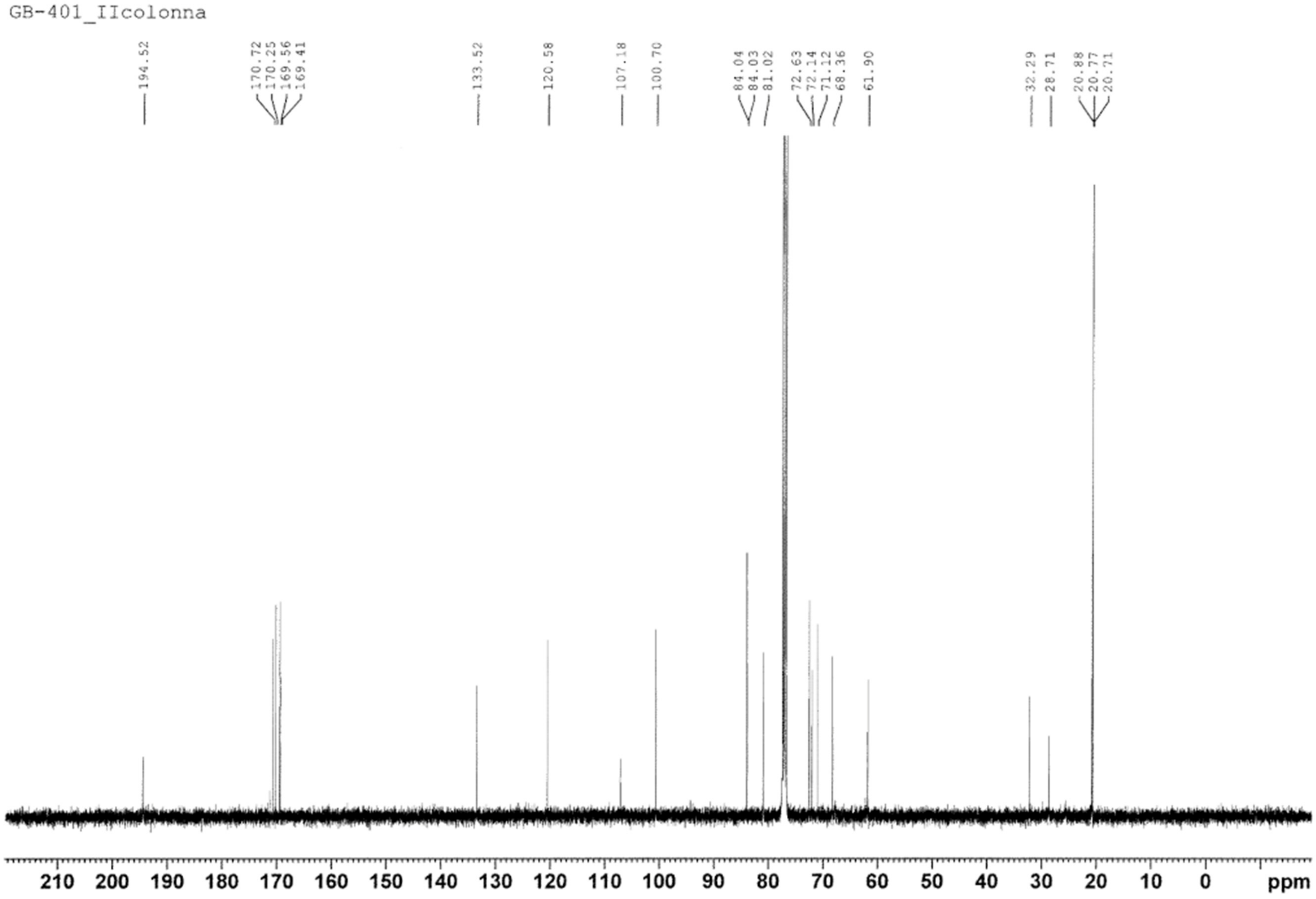

Figure S12. ^{13}C-NMR ($CDCl_3$, 100 MHz) of intermediate **9**.

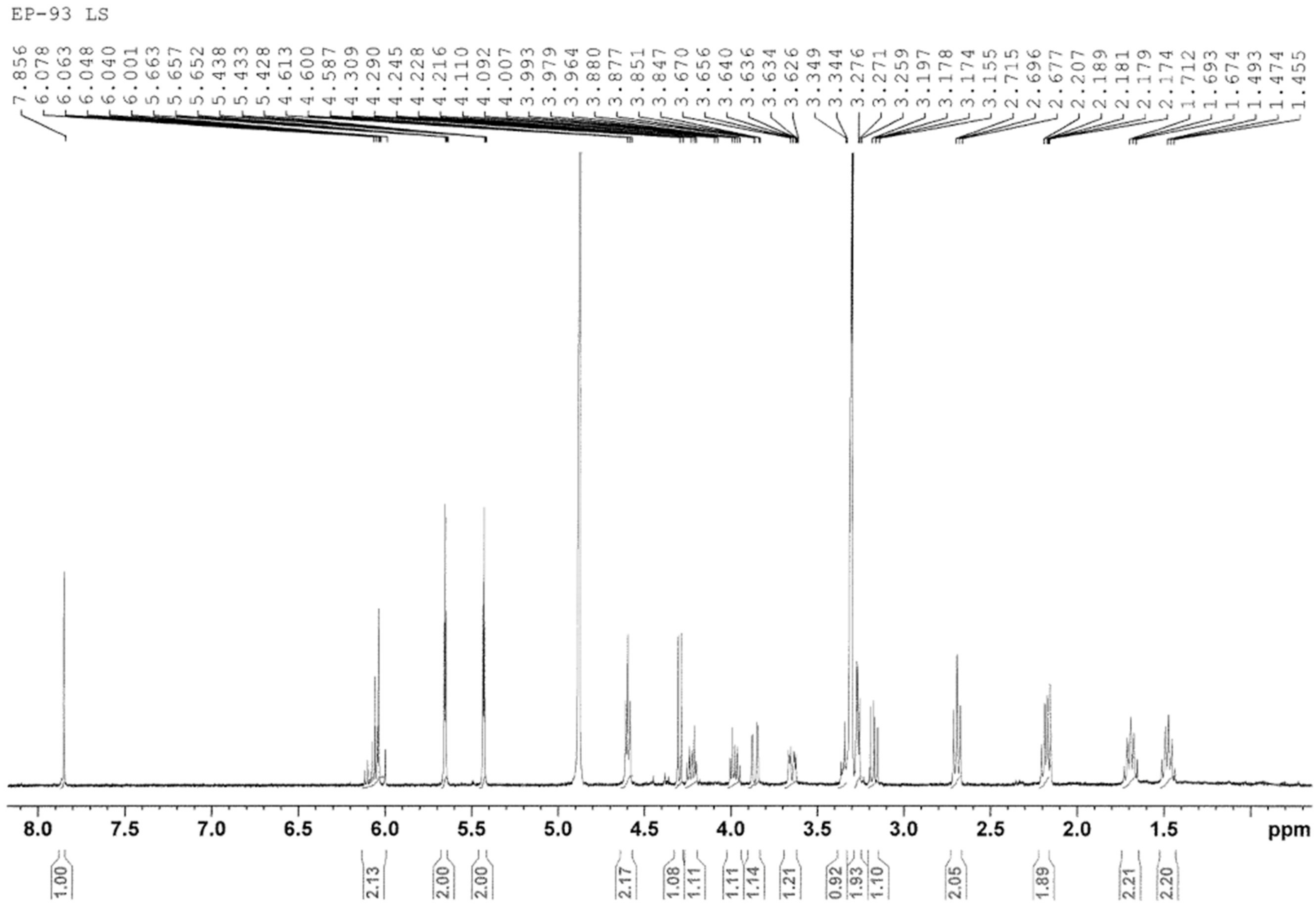

Figure S13. ^{1}H-NMR (CD_3OD, 400 MHz) of intermediate **10**.

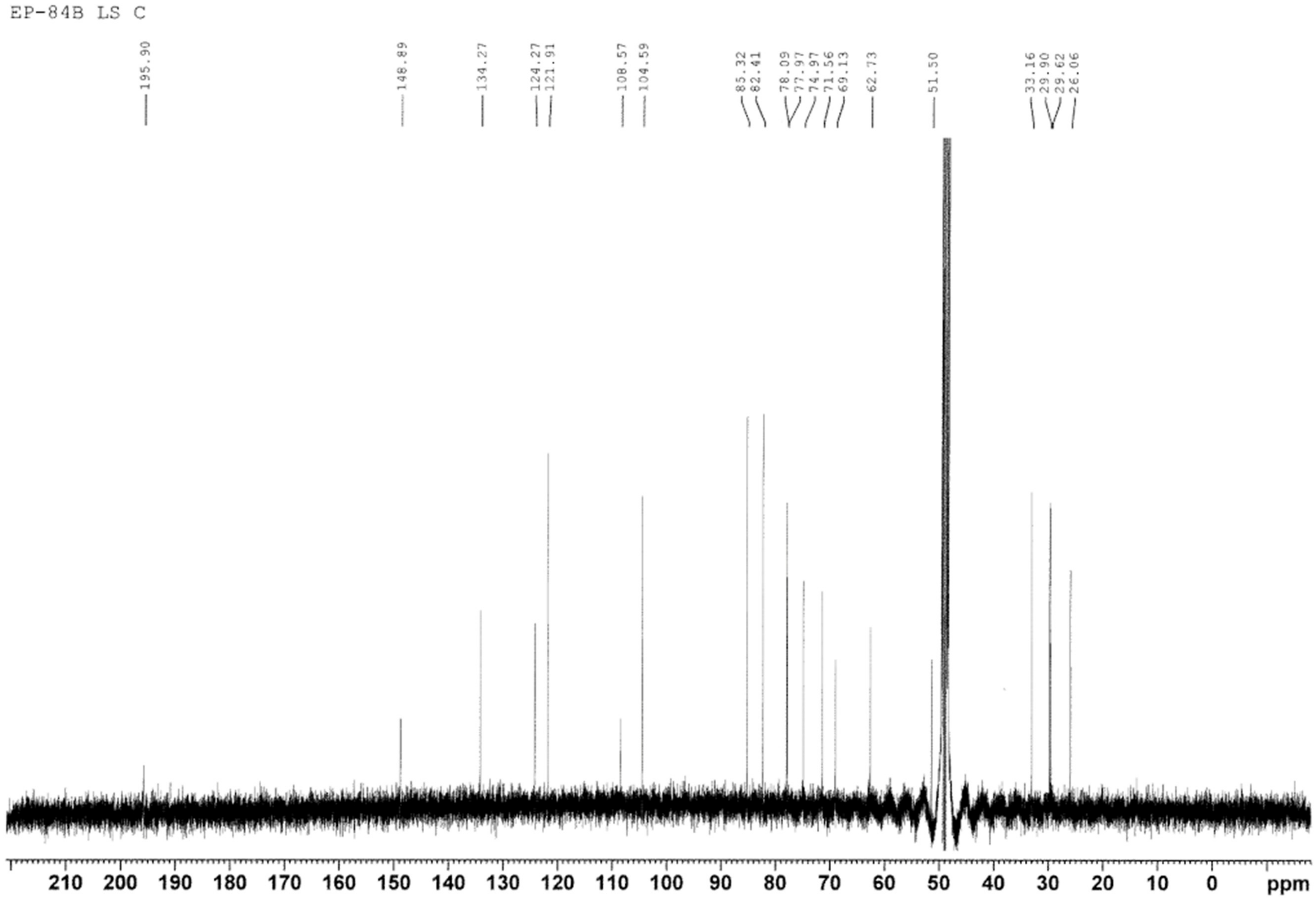

Figure S14. ^{13}C-NMR (CD_3OD, 100 MHz) of intermediate **10.**
